# Smell-related quality of life changes after total laryngectomy: a multi-centre study

**DOI:** 10.1007/s00405-023-07976-0

**Published:** 2023-04-28

**Authors:** Eugene Wong, Murray Smith, Malcolm A. Buchanan, Akshay Kudpaje, Andrew Williamson, Prasanna Suresh Hegde, Daniel Hazan, Jordan Idaire, Mark C. Smith, Niranjan Sritharan, Carsten Palme, Faruque Riffat

**Affiliations:** 1 Department of Otolaryngology, Head and Neck Surgery, Westmead Hospital, University of Sydney, Camperdown, NSW 2006 Australia; 2 Department of Surgery, School of Medicine and Health Sciences, University of Sydney, Camperdown, Australia; 3 Department of ENT Surgery, NHS Greater Glasgow and Clyde, Glasgow, UK; 4 Department of Head and Neck Surgical Oncology, Cytecare Cancer Hospitals, Bangalore, India; 5 Department of Deglutology & Speech Pathology, HCG Hospital, Bengaluru, India; 6 School of Medicine, Western Sydney University, Sydney, Australia; 7 Chris O’Brien Lifehouse, Camperdown, NSW 2006 Australia; 8 Department of Otolaryngology, Head and Neck Surgery, Macquarie University Hospital, Macquarie Park, Australia

**Keywords:** Laryngectomy, Smell, Quality of life, Multicenter, Anosmia, Hyposmia

## Abstract

**Purpose:**

A total laryngectomy creates an alternate airway for gas exchange that bypasses the upper aerodigestive tract. The subsequent reduction in nasal airflow, and therefore, reduction in deposition of particles to the olfactory neuroepithelium leads to hyposmia or anosmia. The aim of this study was to assess the quality of life impairment conferred by anosmia following laryngectomy and identify any specific patient-related risk factors that are associated with poorer outcomes.

**Methods:**

Consecutive patients with a total laryngectomy presenting for review at three tertiary head and neck services (in Australia, the United Kingdom and India) over a 12-month period were recruited. Patient demographic and clinical data were collected, and each subject completed the validated assessment of self-reported olfactory functioning and olfaction-related quality of life questionnaire (ASOF). Dichotomous comparisons were performed using the student's unpaired t-test for continuous variables (SRP), a chi-squared test for categorical variables, and a Kendall's tau-b for ordinal variables (SOC) to assess for a correlation with poorer questionnaire scores.

**Results:**

A total of 66 laryngectomees (13.4% female; age 65.7 ± 8.6 years) were included in the study. The mean SRP score of the cohort was found to be 15.6 ± 7.4, while the mean ORQ score was noted to be 16.4 ± 8.1. No other specific risk factors associated with poorer quality of life were identified.

**Conclusion:**

A significant quality of life detriment from hyposmia is conferred following laryngectomy. Further research to assess treatment options and the patient population that would best benefit from these interventions is required.

## Introduction

A total laryngectomy is a surgical procedure in performed and managed in tertiary head and neck centres which results in bypass of the upper airway during respiration. Total laryngectomy is typically performed in patients with large head and neck cancers involving the larynx. However, other indications for laryngectomy include intractable laryngeal aspiration, severe laryngeal trauma or following severe radiation necrosis [1, 2].

During a laryngectomy an alternate airway is produced where a surgical tracheal stoma to the skin is formed. As a result, no passive airflow passes through the nasal cavity during normal respiration, resulting in several sequelae. The most oft cited is the impairment of the noses' function to humidify, filter and warm inspired air, and as a result patients frequently use a heat-moisture exchanger (HME), nebulisers or humidified oxygen systems (AIRVO) as a surrogate to prevent airway drying and crusting [3–5].

However, the nose is also the primary organ of olfaction. Olfactory neuroepithelium is located high in the nasal cavity, incorporating the superior nasal septum, cribriform plate, superior turbinate and the superolateral nasal wall [6]. In the normal nose (with normal nasal airflow), this epithelium receives only 10 to 15% of total nasal airflow [7]. The axons of these receptor cells then extend into the olfactory bulb before they are transmitted to the olfactory cortex and amygdala.

It therefore follows that following significant reduction or absence of nasal airflow such as in a laryngectomy, odour baring air volume to this area is drastically reduced. Additionally, previous studies have suggested long-term reduction in stimulation of the neuroepithelium result in degeneration, and a reduction in total olfactory bulb volumes [8]. Published studies in the literature certainly support this phenomenon, with hyposmia or anosmia reported to be experienced by 35–78% of patients after laryngectomy [9–11] with a subgroup complaining also of associated gustatory disturbance.

Olfaction serves several important functions that impact on a patient’s quality of life [12]. It facilitates the recognition and enjoyment of food, assists in reproduction and sexual drive (pheromones), and is an important primary defence mechanism and the maintenance of personal hygiene (e.g. detection of rotten food, fire or noxious chemicals; triggering of memories). The impact of hyposmia in significantly impairment of a patient’s quality of life is well documented in the literature [13].

The aim of this study was to determine the specific quality of life detriment caused by hyposmia or anosmia using validated self-reported questionnaires in a cohort of laryngectomees managed at three large tertiary head and neck units around the world – Australia, India, and the United Kingdom. A secondary aim was to identify specific patient-related risk factors the predicted for greater QoL detriment.

## Methods

### Patient population

Consecutive patients who underwent a previous total laryngectomy who presented for routine clinical surveillance review between 1st August 2019 to 1st December 2019 at tertiary head and neck units in Sydney, Australia, (Westmead Hospital), Glasgow and Clyde, UK (NHS) and Bengaluru, India (HCG Hospitals) were invited to participate in the study. Patients were only included if they had a minimum of 12 months of follow-up and underwent a total laryngectomy: i.e. partial laryngectomy cases were excluded.

Following an informed consent process, agreeable patients were recruited into the study following ethics approval from the Western Sydney Local Health District Human Research Ethics Committee (WSLHD HREC.)

### Patient demographic data

Patient demographic, medical, and surgical data were retrieved from medical records held at each of the study institutions. Data retrieved included age, sex, diagnosis and indication for the procedure, date of the procedure, and other specific significant past medical history, where available.

Specific past medical history data collected included diabetes, hypertension, ischaemic heart disease, gastro-oesophageal reflux disease, and chronic airways limitation disease. Diabetes was defined as type 2 diabetes diagnosed by an oral glucose tolerance test or by a specialist endocrinologist and currently medicated with oral anti-hyperglycaemic agents or insulin. Hypertension was defined as a greater than 12-month history of at least one regular antihypertensive medication, and gastro-oesophageal reflux disease was defined as greater than 12-month history of proton-pump inhibitor use. Patients were determined to have ischaemic heart disease if they were diagnosed by a cardiologist, or previously underwent previous percutaneous coronary intervention or coronary artery bypass grafting. Chronic airways limitation was defined as a patient diagnosed by a respiratory physician and confirmed to have a significant smoking history having previous spirometry with a FEV1/FVC of less than 0.7 and an FEV1 < 0.8 of the predicted value based on height, weight and race.

### Assessment of hyposmia severity and effect on quality of life

At the clinical review, the Assessment of Self-reported Olfactory Functioning and olfaction-related quality of life (ASOF) questionnaire [14], was completed by each patient. The ASOF questionnaire is a previously described, 12-item questionnaire consisting of three domains: a subjective olfactory capability scale (SOC), a five-item self-reported capability of perceiving specific odours scale (SRP), and six-item olfactory related quality of life (ORQ) scale. This scale was demonstrated to reliably differentiate patients with olfactory dysfunction with normal controls.

### Statistical analysis

Statistical analysis was performed using the SPSS version 25 (SPSS Inc, IBM, Chicago IL.) Categorical and ordinal descriptive data was presented as percentages with raw numbers. Continuous parametric variables were presented as means with standard deviations. The SOC was considered an ordinal variable while the pooled SRP scores were considered as a single continuous variable.

Dichotomous comparisons were performed using the student's unpaired *t*-test for continuous variables (SRP), a chi-squared test for categorical variables, and a Kendall's tau-b for ordinal variables (SOC.)

Statistical significance was determined at the *p* < 0.05 level.

## Results

### Patient demographics data

A total of 66 patients (13.4% female; age 65.7 ± 8.6 years) were included in the study. Most patients were recruited from India (44.8%), followed by Australia (28.4%) and the United Kingdom (26.9%).

All patients had their procedure performed for tumour resection. Almost all patients had a diagnosis of squamous cell carcinoma (SCC) of the larynx or hypopharynx (98.4%). The remaining patient had a diagnosis of a high-grade chondrosarcoma. Of these, few were performed as a salvage procedure following recurrence from organ preservation therapy (6.0%).

Several patients had significant concurrent comorbidities. The most common comorbidity noted was CAL (14.9%), followed by hypertension (10.4%), T2DM (10.4%) and IHD (10.4%) and finally GORD (9.0%).

Full demographic data are reported in Table 1.

**Table 1:** Full demographic data of all laryngectomees

Demographic	
*N*	66 patients
Age (mean ± SD) years	65.7 ± 8.6 years
% Female	13.4%
*Indication for surgery* (%)	
Squamous cell carcinoma	98.4
Chondrosarcoma	1.6
Salvage procedure (%)	6.0
*Comorbidities* (%)	
Chronic airways limitation	14.9
Hypertension	10.4
Ischaemic heart disease	10.4
Type 2 diabetes	10.4
Gastro-oesophageal reflux disease	9.0

*SD* Standard deviation

### Overall quality of life detriment

Quality of life was significantly impaired in laryngectomy patients across all domains.

72% of patients reported an SOC score of 5 or less, while more than 50% reported an SOC score of 2 or less.

The mean SRP score of the cohort was found to be 15.6 ± 7.4 out of a maximum score of 25 points, while the mean ORQ score was noted to be 16.4 ± 8.1 out of a maximum of 30 points.

Complete breakdown of the individual components of the SRP and ORQ is demonstrated in Table 2.

**Table 2:** Proportion of patients that reported at least “sometimes” having problems (SRP); or areas where they have been at least “moderately impaired” (ORQ) for each subdomain

Question	% scoring “3” or less
*SRP* (%)	
Smelling the odour of spoiled food	52.3
Perceiving your body odour	50.0
Perceiving unpleasant ambient odours (e.g. smoke, gas)	56.7
Perceiving the body odour of women	43.1
Perceiving the body odour of men	42.2
*ORQ* (%)	
Cooking	67.2
Sexual life	59.6
Eating food	58.5
Drinking beverages	52.3
Using perfumes, deodorants, etc	54.8
Perceiving the scent of flowers	67.7

*SRP*  self-reported capability of perceiving specific odours scale, *ORQ*  olfactory related quality of life

### Comparison of risk factors with reduced olfaction-related quality of life

The patient’s country of treatment was not associated with a statistically significant difference for SRP (One-way ANOVA, F = 1.93 with 2df, *p* = 0.16) or ORQ (One-way ANOVA, F = 1.84 with 2df, *p* = 0.17). Age was not significantly correlated with an increase in SRP or ORQ score (Pearson's correlation coefficient 0.01 and  – 0.13, respectively.)

There was no association between a lower SOC score and female sex (Kendall’s tau-B; *p* = 0.11); diabetes (*p* = 0.24); hypertension (*p* = 0.24); ischaemic heart disease (*p* = 0.24); GORD (*p* = 0.83) or CAL (*p* = 0.52).

Similarly, there was also no association for poorer SRP score for female sex (Student’s unpaired *t*-test, 15.8 ± 6.0 vs 15.4 ± 7.6, *p* = 0.90), diabetes (13.6 ± 8.0 vs 16.4 ± 7.3, *p* = 0.37), hypertension (17.4 ± 7.9 vs 15.5 ± 7.4, *p* = 0.55), ischaemic heart disease (14.3 ± 8.3 vs 16.3 ± 7.3, *p* = 0.53), GORD (13.7 ± 8.7 vs 16.3 ± 7.3, *p* = 0.43) or CAL (14.9 ± 7.9 vs 16.3 ± 7.4, *p* = 0.63.)

Similarly there was no association for poorer ORQ score for the same risk factors (Student’s unpaired *t*-test; female sex: 19.1 ± 8.7 vs 16.0 ± 8.0, *p* = 0.29; diabetes: 9.9 ± 6.5 vs 14.8 ± 6.8, *p* = 0.09; hypertension: 14.5 ± 7.7 vs 13.7 ± 6.9, *p* = 0.81; ischaemic heart disease: 12.2 ± 5.2 vs 14.2 ± 7.3, *p* = 0.52; GORD: 12.5 ± 6.3 vs 14.1 ± 7.1, *p* = 0.61; CAL: 13.6 ± 4.9 vs 14.0 ± 7.7, *p* = 0.89.)

## Discussion

A total laryngectomy carries sequelae that can significantly impact a patient's quality of life. These deleterious effects are typically secondary to the loss of the normal physiologic function provided by the upper aerodigestive tract. Olfaction is one such function of the nasal cavity that is significantly disrupted in laryngectomees. We describe, to our knowledge, the largest multi-centre cohort of laryngectomees assessed using a validated hyposmia quality of life measurement tool.

It is our observation that this morbidity is inadequately understood by patients and carers, and equally inadequately addressed in pre-operative workup by clinicians. Treatment information documents provided make, at best, a cursory mention of hyposmia. Our paper highlights the degree and effects of olfactory dysfunction contributing to QOL compromise in laryngectomees, and we hope highlight the importance of addressing this sensory loss.

Patients with olfactory disorders have been found to be impaired in food intake, personal safety, hygiene, sense of well-being and their sexual life. These findings are comparable with Deems' study [15] of 750 patients that found that more than 68% of patients experienced an altered quality of life – and specifically 46% experienced a loss of weight through reduced appetite and 56% reported negative effects on daily living and psychological well-being.

This study demonstrates that the impact of olfactory dysfunction following a laryngectomy also exists in this population, and carries a substantial quality of life burden across several domains. The simple fact that these operations were, in most cases life-saving or life-prolonging, did not cause olfactory-related quality of life disturbance to be better tolerated or accepted by the patient population. Additionally, this study has suggested that laryngectomees, regardless of their age, sex cultural, geopolitical and socioeconomic background and past medical history are affected equally by post-operative anosmia.

Our multinational three centre study also allowed us to compare cultural and dietary impact of olfactory dysfunction. Despite the expectation of differences, given some cultural diets are composed of spices that provide greater odorous stimulation, we found none.

Nevertheless, it is the author’s opinion that patients where hyposmia is noted as a significant material risk or whose daily activities depend on smell, such as a firefighter, cook, or wine merchant, would be expected to experience a more significant reduction in quality of life, and therefore should be emphasised specifically during the consent process and in the management of expectations. In equal measure, one might highlight cultural practices where communal multigeneration dining is the norm, the degree of social isolation felt by the laryngectomee, already impaired of speech would be compounded.

The olfactory process has been described as both containing passive, which occurs during normal nasal breathing, and active processes during an active sniff [16]. While passive smelling in laryngectomy patients is often heavily impacted, active smelling may be retained to a limited degree. This provides a vital avenue for training and minimization of disability from sensory loss. One may even consider training pre-operatively by speech pathologists as early implementation of deliberate olfaction may lessen the expected sensory degeneration.

Rehabilitation of olfaction after these procedures have previously been described that incorporate maximal use of active smelling, such as in a "polite yawning" technique, however, the overall efficacy or effect on quality of life remains controversial [17–19]. Nevertheless, it may follow that olfactory rehabilitation therapy should be initiated as soon as possible to mitigate the effects of olfactory neurodegeneration as previously described [20].

Several limitations exist with this study. Firstly, the cohort sized included was small, as the questionnaire data were filled prospectively. Additionally, the premorbid impact on hyposmia, or the prevalence of other disease factors that may confound results, such as conditions causing nasal obstruction or neurodegenerative disorders known to cause hyposmia was not assessed. Nevertheless, this study, to our knowledge, represents the largest cohort of laryngectomees assessed using a validated hyposmia quality of life measurement tool.

Secondly, this study only included a single cohort of patients. Additionally, all patients in our study underwent laryngectomy malignancy, and almost ubiquitously with a diagnosis of squamous cell carcinoma. As a result, this study is unable to provide any comparisons to a control cohort of patients without laryngectomy, or patients who underwent laryngectomy for other indications, although one would surmise that disability in non-SCC would be similar given evidentiary airflow alteration. Furthermore, given our patient cohort includes both primary and salvage cases, we are unable to comment on impact of chemoradiation.

Nevertheless, given that a significant quality of life detriment from olfactory loss is established, a number of future directions for research are apparent. As aforementioned, while a number of techniques for smell rehabilitation have been described, it is critical that large cohort studies assessing their positive impact on quality of life need to be established. Then, any predictive factors for poorer outcome in future studies may be used to allow at-risk populations to have smell rehabilitation integrated early in their multidisciplinary treatment plan, or alternatives such as partial laryngectomy and/or chemoradiotherapy may be considered with greater emphasis in patients where olfactory loss would present a material change to QOL and occupation.

## Conclusion

This study demonstrates that patients who undergo total laryngectomy experience a sustained and significant quality of life detriment from hyposmia. Further research to assess the optimal treatment and management options, as well as to define the appropriate patient population that would best benefit from these interventions is suggested.

## Data Availability

The data to support the findings of this study are available from the corresponding author E.W upon request.

## References

[1] Chin OY, Dubal PM, Sheikh AB, Unsal AA, Park RCW, Baredes S et al (2017) Laryngeal chondrosarcoma: a systematic review of 592 cases. Laryngoscope 127(2):430–439 27291822 10.1002/lary.26068

[2] Hutcheson KA, Alvarez CP, Barringer DA, Kupferman ME, Lapine PR, Lewin JS (2012) Outcomes of elective total laryngectomy for laryngopharyngeal dysfunction in disease-free head and neck cancer survivors. Otolaryngol Head Neck Surg 146(4):585–590 22235071 10.1177/0194599811432264 PMC4005839

[3] Mérol J-C, Charpiot A, Langagne T, Hémar P, Ackerstaff AH, Hilgers FJ (2012) Randomized controlled trial on postoperative pulmonary humidification after total laryngectomy: External humidifier versus heat and moisture exchanger. Laryngoscope 122(2):275–281 22105893 10.1002/lary.21841

[4] Wong C, Shakir A, Farboud A, Whittet H (2016) Active versus passive humidification for self-ventilating tracheostomy and laryngectomy patients: a systematic review of the literature. Clin Otolaryngol 41(6):646–651 26505471 10.1111/coa.12577

[5] Birk M, Bauerfeind P, Deprez PH, Häfner M, Hartmann D, Hassan C et al (2016) Removal of foreign bodies in the upper gastrointestinal tract in adults: European society of gastrointestinal endoscopy (esge) clinical guideline. Endoscopy 48(05):489–496 26862844 10.1055/s-0042-100456

[6] Leopardi G, Chiarella G, Conti S, Cassandro E (2008) Surgical treatment of recurring preauricular sinus: supra-auricular approach. Acta Otorhinolaryngol Ital 28(6):302–305 19205595 PMC2689545

[7] Hornung DE (2006) Nasal anatomy and the sense of smell. Taste Smell 63:1–22 10.1159/000093747 16733330

[8] Veyseller B, Ozucer B, Aksoy F, Yildirim YS, Gürbüz D, Balikçi HH et al (2012) Reduced olfactory bulb volume and diminished olfactory function in total laryngectomy patients: a prospective longitudinal study. Am J Rhinol Allergy 26(3):191–193 22643943 10.2500/ajra.2012.26.3768

[9] Ackerstaff A, Hilgers F, Aaronson N, Balm A (1994) Communication, functional disorders and lifestyle changes after total laryngectomy. Clin Otolaryngol Allied Sci 19(4):295–300 7994884 10.1111/j.1365-2273.1994.tb01234.x

[10] Van Dam FS, Hilgers FJ, Emsbroek G, Touw FI, Van As CJ, De Jong N (1999) Deterioration of olfaction and gustation as a consequence of total laryngectomy. Laryngoscope 109(7):1150–1155 10401859 10.1097/00005537-199907000-00027

[11] Risberg-Berlin B, Ylitalo R, Finizia C (2006) Screening and rehabilitation of olfaction after total laryngectomy in Swedish patients: Results from an intervention study using the nasal airflow–inducing maneuver. Arch Otolaryngol Head Neck Surg 132(3):301–306 16549751 10.1001/archotol.132.3.301

[12] Miwa T, Furukawa M, Tsukatani T, Costanzo RM, DiNardo LJ, Reiter ER (2001) Impact of olfactory impairment on quality of life and disability. Arch Otolaryngol Head Neck Surg 127(5):497–503 11346423 10.1001/archotol.127.5.497

[13] Boesveldt S, Postma EM, Boak D, Welge-Luessen A, Schopf V, Mainland JD et al (2017) Anosmia-a clinical review. Chem Senses 42(7):513–523 28531300 10.1093/chemse/bjx025 PMC5863566

[14] Pusswald G, Auff E, Lehrner J (2012) Development of a brief self-report inventory to measure olfactory dysfunction and quality of life in patients with problems with the sense of smell. Chemosens Percept 5(3–4):292–299

[15] Deems DA, Doty RL, Settle RG, Moore-Gillon V, Shaman P, Mester AF et al (1991) Smell and taste disorders, a study of 750 patients from the University of Pennsylvania Smell and Taste Center. Arch Otolaryngol Head Neck Surg 117(5):519–528 2021470 10.1001/archotol.1991.01870170065015

[16] Wachowiak M (2011) All in a sniff: olfaction as a model for active sensing. Neuron 71(6):962–973 21943596 10.1016/j.neuron.2011.08.030 PMC3237116

[17] Hilgers FJ, van Dam FS, Keyzers S, Koster MN, van As CJ, Muller MJ (2000) Rehabilitation of olfaction after laryngectomy by means of a nasal airflow-inducing maneuver: the polite yawning technique. Arch Otolaryngol Head Neck Surg 126(6):726–732 10864109 10.1001/archotol.126.6.726

[18] Risberg-Berlin B, Möller RY, Finizia C (2007) Effectiveness of olfactory rehabilitation with the nasal airflow-inducing maneuver after total laryngectomy: One-year follow-up study. Arch Otolaryngol Head Neck Surg 133(7):650–654 17638776 10.1001/archotol.133.7.650

[19] Moor J, Rafferty A, Sood S (2010) Can laryngectomees smell? Considerations regarding olfactory rehabilitation following total laryngectomy. J Laryngol Otol 124(4):361–365 20059791 10.1017/S0022215109992489

[20] Miani C, Ortolani F, Bracale AM, Petrelli L, Staffieri A, Marchini M (2003) Olfactory mucosa histological findings in laryngectomees. Eur Arch Otorhinolaryngol 260(10):529–535 12835945 10.1007/s00405-003-0638-3

