# Correction: Smell-related quality of life changes after total laryngectomy: a multi-centre study

**DOI:** 10.1007/s00405-023-08161-z

**Published:** 2023-08-16

**Authors:** Eugene Wong, Murray Smith, Malcolm A. Buchanan, Akshay Kudpaje, Andrew Williamson, Prasanna Suresh Hedge, Daniel Hazan, Jordan Idiare, Mark C. Smith, Niranjan Sritharan, Carsten Palme, Faruque Riffat

**Affiliations:** 1 Department of Otolaryngology, Head and Neck Surgery, Westmead Hospital, University of Sydney, Camperdown, NSW 2006 Australia; 2 Department of Surgery, School of Medicine and Health Sciences, University of Sydney, Camperdown, Australia; 3 Department of ENT Surgery, NHS Greater Glasgow and Clyde, Glasgow, UK; 4 Department of Head and Neck Surgical Oncology, Cytecare Cancer Hospitals, Bangalore, India; 5 School of Medicine, Western Sydney University, Sydney, Australia; 6 Chris O’Brien Lifehouse, Camperdown, NSW 2006 Australia; 7 Department of Otolaryngology, Head and Neck Surgery, Macquarie University Hospital, Macquarie Park, Australia

**Correction: European Archives of Oto-Rhino-Laryngology (2023) 280:3861–3866** 10.1007/s00405-023-07976-0

In the original publication of the article, the following author’s “Jordan Idiare” last name was published incorrectly as “Idaire”. The name is corrected in this correction article.

