# Correction: Smell-related quality of life changes after total laryngectomy: a multi-centre study

**DOI:** 10.1007/s00405-026-10181-4

**Published:** 2026-06-20

**Authors:** Eugene Wong, Murray Smith, Malcolm A. Buchanan, Akshay Kudpaje, Andrew Williamson, Prasanna Suresh Hegde, Daniel Hazan, Jordan Idaire, Mark C. Smith, Niranjan Sritharan, Carsten Palme, Faruque Riffat

**Affiliations:** 1 Department of Otolaryngology, Head and Neck Surgery, Westmead Hospital, University of Sydney, Camperdown, 2006 NSW Australia; 2 Department of Surgery, School of Medicine and Health Sciences, University of Sydney, Camperdown, Australia; 3 Department of ENT Surgery, NHS Greater Glasgow and Clyde, Glasgow, UK; 4 Department of Head and Neck Surgical Oncology, Cytecare Cancer Hospitals, Bangalore, India; 5 Department of Deglutology & Speech Pathology, HCG Hospital, Bengaluru, India; 6 School of Medicine, Western Sydney University, Sydney, Australia; 7 Chris O’Brien Lifehouse, Camperdown, 2006 NSW Australia; 8 Department of Otolaryngology, Head and Neck Surgery, Macquarie University Hospital, Macquarie Park, Australia

**Correction: European Archives of Oto-Rhino-Laryngology (2023) 280:3861-3866**

10.1007/s00405-023-07976-0

In this article, the author’s name 'Prasanna Suresh Hegde' was incorrectly written as 'Prasanna Suresh Hedge'.

The affiliation details for Prasanna Suresh Hegde were incorrectly given as 'Department of Head and Neck Surgical Oncology, Cytecare Cancer Hospitals, Bangalore, India' but should have been 'Department of Deglutology & Speech Pathology, HCG Hospital, Bengaluru'.

The original article has been corrected.

